# A Comparative Analysis of the Corrosive Effect of Artificial Saliva of Variable pH on DMLS and Cast Co-Cr-Mo Dental Alloy

**DOI:** 10.3390/ma7096486

**Published:** 2014-09-11

**Authors:** Tatjana Puskar, Danimir Jevremovic, Robert J. Williams, Dominic Eggbeer, Djordje Vukelic, Igor Budak

**Affiliations:** 1 Department of Dentistry, Medical Faculty, University of Novi Sad, Hajduk Veljkova 3, 21000 Novi Sad, Serbia; 2 Clinic for Prosthodontics, School of Dentistry, University Business Academy, Zarka Zrenjanina 179, 13000 Pancevo, Serbia; E-Mail: dr.danimir@sbb.rs; 3 Centre for Dental Technology, Cardiff Metropolitan University, PDR UWIC Western Avenue, Cardiff CF5 2YB, UK; E-Mail: rjwilliams@cardiffmet.ac.uk; 4 National Centre for Product Design and Development Research, Cardiff Metropolitan University, PDR UWIC Western Avenue, Cardiff CF5 2YB, UK; E-Mail: deggbeer-pdr@cardiffmet.ac.uk; 5 Department of Production Engineering, Faculty of Technical Sciences, University of Novi Sad, Trg Dositeja Obradovica 6, 21000 Novi Sad, Serbia; E-Mails: vukelic@uns.ac.rs (D.V.); budaki@uns.ac.rs (I.B.)

**Keywords:** dental alloys, corrosion resistance, direct metal laser sintering (DMLS), Co-Cr-Mo alloy

## Abstract

Dental alloys for direct metal laser sintering (DMLS) are available on the market today, but there is little scientific evidence reported on their characteristics. One of them is the release of ions, as an indicator of the corrosion characteristics of a dental alloy. Within this research, the difference in the elution of metals from DMLS and cast (CM) samples of Co-Cr-Mo dental alloy in saliva-like medium of three different pH was examined by inductively-coupled plasma mass spectrometry (ICP-MS). The obtained results show that the metal elution in artificial saliva from the DMLS alloy was lower than the elution from the CM alloy. The release of all investigated metal ions was influenced by the acidity, both from the DMLS and CM alloy, throughout the investigated period of 30 days. The change in acidity from a pH of 6.8 to a pH of 2.3 for the cast alloy led to a higher increase of the elution of Co, Cr and Mo from CM than from the DMLS alloy. The greatest release out of Co, Cr and Mo was for Co for both tested alloys. Further, the greatest release of all ions was measured at pH 2.3. In saliva of pH 2.3 and pH 4.5, the longer the investigated period, the higher the difference between the total metal ion release from the CM and DMLS alloys. Both alloys showed a safe level of elution according to the ISO definition in all investigated acidic environments.

## 1. Introduction

Cobalt-Chromium-Molybdenum (Co-Cr-Mo) alloy is widely used alloy for the fabrication of removable partial dentures (RPDs) and porcelain-fused-to-metal crowns in dentistry today. It has increasingly replaced noble metal alloys, because of better mechanical properties and lower cost, although non-precious metal alloys are more difficult to cast. Certain inaccuracies may occur in casting Co-Cr-Mo alloys due to their higher melting range, limited ductility and potential for oxidation [1,2]. The time and labour required for traditional casting of Co-Cr-Mo alloy makes fabrication expensive, and efforts have been made to improve the technological process to overcome the shortcomings and to reduce costs.

Direct metal laser sintering (DMLS) is a promising technology that may enable the fabrication of dental devices, overcoming some of the imperfections of casting [3]. The process of DMLS fabrication is an additive manufacturing (AM) process in which 3D parts are fabricated by the layered addition of material directly on the basis of computer aided design (CAD) data. In that way, DMLS enables a quick fabrication of 3D parts of any complex shape [4,5]. A high power laser is used to melt a powder feedstock to form fully-dense metallic parts [6]. Thus, objects fabricated by DMLS are extremely dense, and the mechanical properties are comparable to, or better than, those of cast or machined parts. Accordingly, they can often replace parts produced by traditional methods in many applications [7]. In dental applications, DMLS is a technique that could replace conventional metal casting procedures, as it can be used as a tool for the production of customized dental parts from biocompatible alloys directly.

Dental alloys for DMLS are available on the market today, but there is little scientific evidence reported on their characteristics, although the manufacturers state that the alloys are compliant with the standards of the International Organisation for Standardisation (ISO) [8]. Nevertheless, the data are not fully available for academic scrutiny. It may also be worth noting that the DMLS process itself may affect the mechanical and chemical properties of the alloy. Materials for dental applications have to meet unique requirements, including suitable mechanical properties [9] and acceptable biocompatibility, as they are to be placed in the oral environment with a variable pH [10].

**Figure 1:**
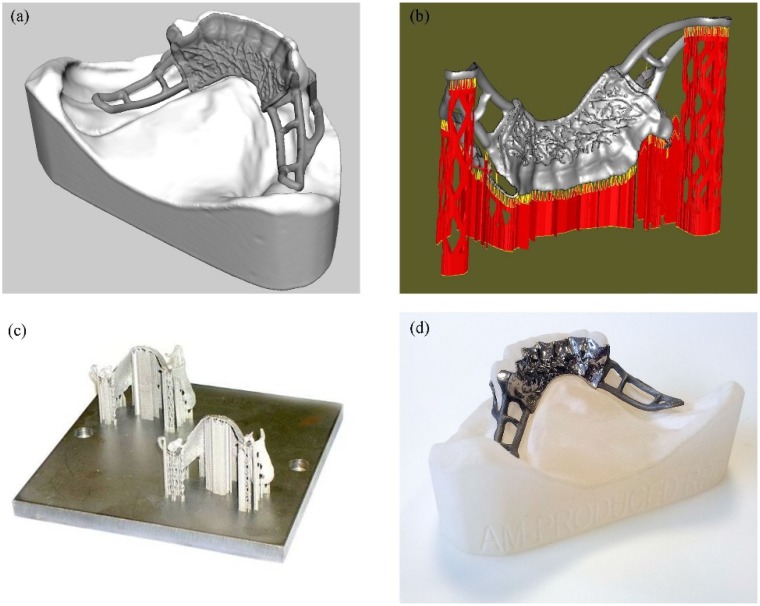
Removable partial denture (RPD) framework; (**a**) virtual design; (**b**) digital model with all relevant features for direct metal laser sintering (DMLS) manufacturing; (**c**) RPM framework fabricated on DMLS; (**d**) the final RPD framework after the finishing test fitted to the original patient cast.

The release of ions from dental devices is an indicator of the corrosion characteristics of dental alloy. The corrosion characteristics, as well as other properties of the alloy are influenced by its microstructure. It is known that the technological procedure has an influence on the structure of the alloy [11,12]. DMLS is a completely different technological procedure from traditional casting that has been used for decades for manufacturing Co-Cr-Mo alloy dental devices [13]. The dental device structure is built by layers created as cross-sections from a 3D CAD file that contains the framework design generated on the virtual model of the patient’s dental arch (Figure 1). It is most important to prove that dental devices manufactured by this new and promising technology are not harmful for the patient.

Both the type of the released element and its quantity have an influence on the corrosion characteristics of an alloy. Many metals, such as chromium (Cr) and cobalt (Co), are toxic, even at low levels of exposure. Although Co is an essential element, most studies emphasize the fact that Co at higher concentrations is toxic and carcinogenic, with oxidative stress playing a crucial role [14]. Experimental evidence points out that hexavalent Cr exposure, by either inhalation or ingestion, can have systemic effects that are distant from the site of exposure. Since hexavalent Cr is isostructural with sulphate and phosphate at physiological pH, it can be carried throughout the body and even into the brain [15]. The previously noted points show the importance of determining the quantity of metal released in the oral cavity from DMLS dental alloy, as a new technology.

Metals released from dental alloys in artificial saliva are present in trace amounts. Their detection should be accomplished using a methodology that has a low detection limit and high specificity. Inductively-coupled plasma mass spectrometry (ICP-MS) is a suitable method for the detection of trace metals. This technique is a type of mass spectrometry with a very low detection limit for metals, which can be lower than one part per trillion (ppt). At certain points in the process of ICP-MS, the temperature is raised to 6000 °C, enough to excite and ionize all metals, which then emit their own spectrum. The concentration of the released ions is calculated according to the intensity of light [16].

The corrosive effect of laser-sintered (LS) Co-Cr-Mo alloy has so far only been investigated by Alifui-Segbaya *et al.* [17], Takaichi *et al.* [12] and Zhang *et al.* [18]. Alifui-Segbaya *et al.* [17] investigated the dissolution of metal ions from rapid manufactured (RM) and cast Co-Cr-Mo alloy in artificial saliva of pH 2.3 and found that RM alloy performed better. Takaichi *et al.* [12] investigated the characteristics of selective laser melted (SLM, another type of metal RM process) Co-Cr-Mo alloy of experimental composition. The dissolution of metals from experimental Co-Cr-Mo SLM alloy in one mass % lactic acid was lower than from the cast Co-Cr-Mo alloy. Zhang Biao *et al.* [18] in a preliminary study on some properties of Co-Cr-Mo dental alloy formed by SLM investigated some of its properties and ion release behaviour after porcelain fused firing. The preliminary result indicated that SLM alloy performed better for Co, but the detection limit was not low enough to detect Cr release in this investigation.

Other researchers that investigated DMLS Co-Cr-Mo alloy focused their interest on the accuracy and misfit of fixed dental restorations, comparing them to cast Co-Cr-Mo devices [13,19,20,21]. The results of these investigations suggest that DMLS (LS) dental devices performed better than the conventional devices, except the investigation of Kim *et al.* [22], who found that the marginal fit of the DMLS system appeared significantly inferior to the conventional method.

The necessity of investigating the ion release from dental alloy in artificial saliva of different acidity exists because the oral cavity is a very demanding environment with high humidity, bacteria presence and variable pH. Bacteria significantly reduce the pH of the oral environment by the production of organic acids during sugar catabolism [23]. To maintain a non-harmful pH in the oral cavity, the salivary system employs buffer systems: bicarbonate, phosphate and protein that maintain a pH of 6.0–7.5 [24]. It is confirmed that high acidity can occur in small entrapped spaces that can be formed near dental devices where the buffering capacity of the saliva is diminished due to the reduced flow (fluctuation) or when gastric juice is ejected from the oesophagus into the oral cavity [25,26].

There is little evidence of the dissolution of metals of DMLS in saliva-like media in the scientific literature, while the influence of pH variations has not yet been reported. The research presented here is a comparative study of the dissolution of metals of two Co-Cr-Mo alloys for a removable partial denture framework, fabricated using DMLS and casting technologies in artificial saliva of three different pH.

## 2. Materials and Method

### 2.1. Specimen Fabrication

Specimens for the investigation of metal release from dental Co-Cr-Mo alloy into the artificial saliva were prepared to simulate the preparation of denture frameworks for clinical cases. DMLS and cast test specimens were prepared in accordance with the ISO 10271 [27] and ISO 22674 [28] specifications. The number of samples in each test group was above the ISO standard, which requires at least two parallel sets.

#### 2.1.1. DMLS Specimen Production

The specimen design was a rectangular prismatic plate of dimensions of 47 mm × 12 mm × 4 mm. The size of a specimen was slightly larger than the final specimen size in order to allow for the reduction of the size to precise dimensions and the elimination of any curling and delaminating that may have occurred during the DMLS fabrication process. On the basis of the CAD data, DMLS specimens were produced on an EOSINT M270 system (EOS GmbH, Robert-Stirling-Ring 1, Krailling, Germany). Processing parameters for the Yb-fibre laser are given in Table 1.

**Table 1:** Processing parameters used for specimen fabrication on the EOSINT M270.

Processing parameter	Part border	Part hatch (core)
Laser power (W)	200	200
Laser point distance (μm)	70	85
Laser exposure time (ns)	50	80
Laser beam spot compensation (mm)	0.079	0.079

**Figure 2:**
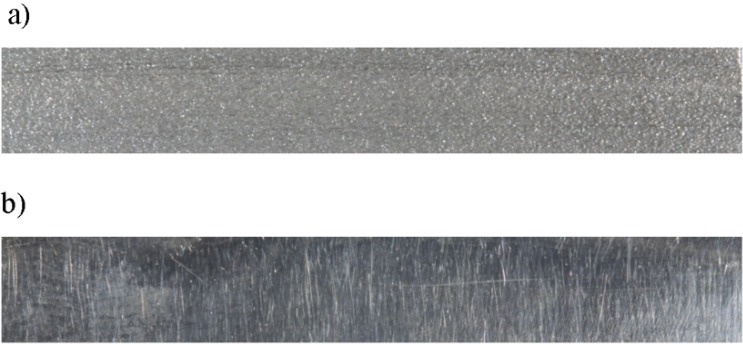
DMLS specimen: (**a**) before polishing; (**b**) after polishing.

The material used for final sample fabrication was SP2 RM type 4 alloy (EOS GmbH). The composition by mass of SP2, according to the manufacturer, in percentages, is as follows: Co 63.8%, Cr 24.7%, Mo 5.1%, W 5.4%, Si 1.0, Fe < 0.5% and Mn < 0.1%. The specimens were finally prepared according to ISO 10271 [27]. The plates were prepared by removing at least 0.1 mm of material from the surfaces using ceramic bonded stones. All specimens were subsequently polished with abrasive rubber wheels and finally polished with Co-Cr-Mo polishing paste (Bego, Bremen, Germany) (Figure 2). Finally, the areas of the samples’ surfaces were measured, and means and standard deviations were calculated for cast (CM) (M = 996.04 mm^2^, SD = 6.69 mm^2^) and DMLS (M = 1001.85 mm^2^, SD = 5.31 mm^2^).

#### 2.1.2. Cast Specimen Production

**Figure 3:**
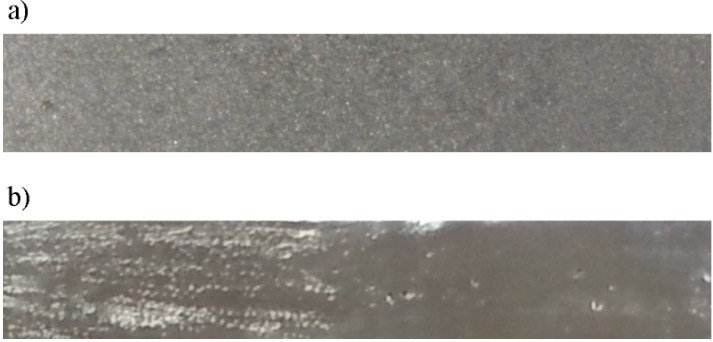
Cast specimen: (**a**) before polishing; (**b**) after polishing.

Sacrificial patterns in the form of rectangular prismatic plates made in acrylic resin (GC Pattern Resin LS, GC Corporation Japan) of dimensions of 46 mm × 11 mm × 3 mm were prepared. The dimensions were measured with a micrometre, according to ISO 10271 [27], and carefully examined for porosity. Afterwards, patterns were spruced with 4 mm diameter wax and invested in phosphate bonded precision investment material for RPD frameworks (Rema dynamic S, Dentaurum, Ispringen, Germany). The powder liquid mixing ratio was 100 g of powder to 16 mL of expansion fluid. The investment muffles were heated gradually in a furnace and cast in a Nautilus CC vacuum pressure casting machine (Bego, Bremen, Germany). Co-Cr-Mo dental alloy Remanium GM 800+ (Dentaurum, Ispringen, Germany) was used. The composition of the alloy by mass according to the manufacturer is given in percentages as follows: Co 63.3%, Cr 30%, Mo 5%, Si 1%, Mn < 1%, W < 1%, N < 0.1% and C < 0.1%. After casting, the muffles were left to cool and the plates were divested and blasted (Figure 3). Final preparation of the specimens was carried out according to ISO 10271 [27], following the same procedure described for the final preparation of DMLS specimens.

### 2.2. Test Procedure of the Dissolution of Metals

**Figure 4:**
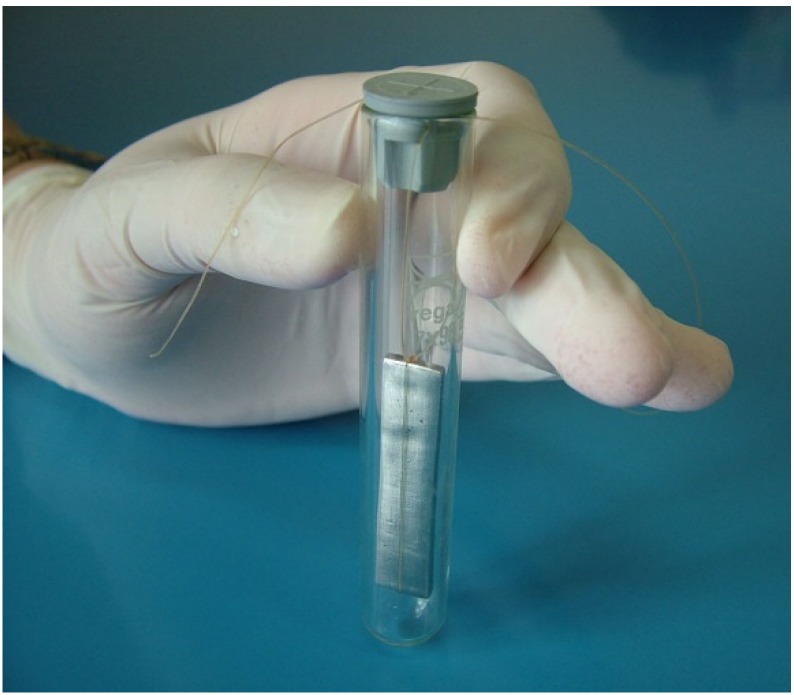
Specimen in glass container suspended on a nylon thread.

Specimens were ultrasonically cleaned for 2 min in ethanol, rinsed with distilled water and dried with oil- and water-free compressed air. Each specimen was placed in a separate glass container and suspending on a nylon thread (Figure 4). The specimens were completely immersed in a solution of artificial saliva (NaCl, Lactic acid) of pH 2.3 ± 0.1, pH 4.0 ± 0.1 and pH 6.8 ± 0.1. The pH of the freshly prepared solutions was measured with a pH-meter (S20-K SevenEasy™ pH, Mettler Toledo, Columbus, OH, USA) before immersion of specimens and after each test period. The glass containers were sealed to prevent evaporation and maintained at 37 °C in a water bath for the testing period. The testing period was 1, 7, 14 and 30 days. The quantity of the artificial saliva in every glass container was such to produce a ratio of 1 mL of solution per 1 cm^2^ of sample surface area. The volume of solution was recorded to an accuracy of 0.1 mL. Additional glass containers were used for two reference solutions without immersed specimens for each test group. They were tightly sealed and maintained in the same conditions with the solutions containing the specimens until the end of a test period.

A total of 24 different glass containers were prepared. At the end of each testing period, a sample of solution was taken and tested in an ICP-MS (Nexion 300X PerkinElmer, Waltham, MA, USA) in a reaction mode (oxygen). ICP-MS used for detection of Co, Cr and Mo release is currently considered one of the most successful analytical methods [29,30]. The advantages of ICP-MS overcome all of the shortcomings of conventional methods, making the analysis simple and controllable. With conventional methods, trace elements could not be detected. When working with a complex material, the matrix could influence the accuracy of the results, and the linearity of the conventional methods could not be presented in as wide of a concentration range as with ICP-MS (from ppt to ppm) [16]. The ICP-MS technique provides excellent sensitivity, requires minimal sample size, affords minimal elemental interferences and provides a means to perform rapid and automated multi-elemental analyses, and there is no dependence of the various chemical functionalities contained in the sample matrices on the individual element recoveries. Running in reaction mode, ICP-MS enabled detection of Cr with the ultimate detection limit, as Cr is difficult to detect, due to interferences [16].

### 2.3. Density Measurement

Density measurement was performed according to instructions from ISO 22674 [28]. Five specimens of CM and DMLS were immersed in ethanol and cleaned in an ultrasonic bath (Renfert EASY CLEAN, Renfert GmbH, Germany) for 2 min. Afterwards, the specimens were rinsed with distilled water and dried with water-free and oil-free compressed air (Sirona C8+ dental unit, Sirona Dental Salcburg, Austria). For density determination, a pycnometer (Rajas Enterprises, Haryana, India) was used.

### 2.4. Microstructure Analyses

The microstructure of CM and DMLS Co-Cr-Mo alloys was analysed by inverted microscope (Olympus GX51, Tokyo, Japan). For this purposes, special samples—rectangular plates (10 mm × 10 mm × 1.5 mm)—were fabricated, also according to standard ISO 22674 [28]. After preparation, which was comprised of polishing, rinsing (in distilled water and in 97% ethanol) and air drying, the surfaces of specimens were etched with a solution that consisted of HCl, 30% H_2_O_2_ and concentrated H_2_SO_4_ in a proportion of 3:1:1. The microstructure was analysed on samples’ cross sections.

## 3. Results and Discussion

**Figure 5:**
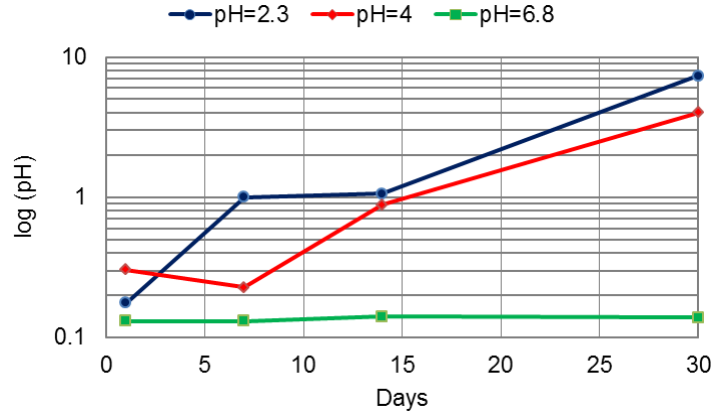
The difference between the release of Co from the CM and DMLS alloys (μg per cm^2^) in artificial saliva of pH = 2.3, pH = 4 and pH = 6.8.

The released Co from the CM and DMLS alloys in artificial saliva of pH = 2.3, pH = 4 and pH = 6.8 in μg per cm^2^ is presented in Table 2. The release of Co was the highest compared to the release of Cr and Mo, both from the CM and DMLS alloys. The highest release of Co was found in the most acidic environment. The difference between the release of Co from the CM and DMLS alloys is shown in Figure 5. The greatest difference was measured after 30 days of immersion at pH of 2.3. The Co release was 7 μg per cm^2^ more from the CM alloy than from the DMLS alloy. The released quantity of Co from the DMLS alloy compared to the cast alloy at pH of 2.3 was 1.5-times less after one day, about 2.5-times less after seven and 14 days and 10-times less after 30 days of immersion (Table 2, Figure 5). At a pH of 4, the release of Co from the DMLS alloy was two- to four-times lower compared to the cast alloy, while at a pH of 6.8, the release was 3.8- to 5.8-times lower.

**Figure 6:**
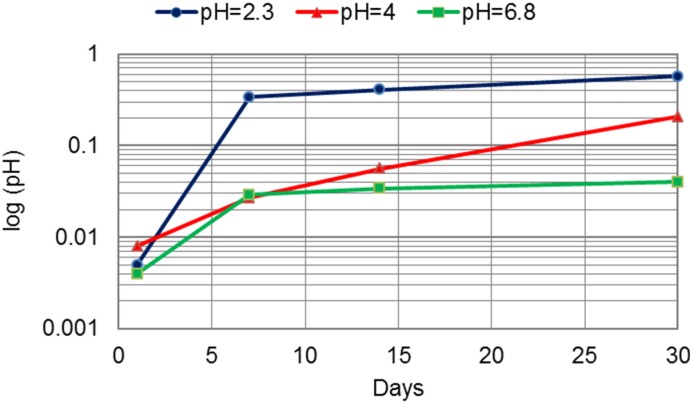
The difference between the release of Cr from the CM and DMLS alloys (μg per cm^2^) in artificial saliva of pH = 2.3, pH = 4 and pH = 6.8.

**Figure 7:**
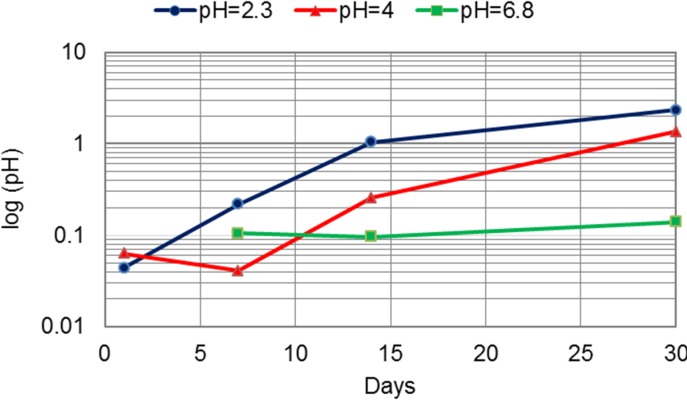
The difference between the release of Mo from the CM and DMLS alloys (μg per cm^2^) in artificial saliva of pH = 2.3, pH = 4 and pH = 6.8.

The release of Cr was the lowest compared to all other measured metal release (Table 3). The difference between the release from the CM and DMLS alloys was the highest after 30 days of immersion in the most acid environment for Cr (Figure 6) and Mo (Table 4, Figure 7).

**Table 2:** The released Co from the cast (CM) and DMLS alloys (μg per cm^2^) in artificial saliva of pH = 2.3, pH = 4 and pH = 6.8.

pH	Sample	Time of the Exposure			
		1 Day	7 Days	14 Days	30 Days
2.3	CM	0.500	1.685	1.819	8.244
	DMLS	0.324	0.685	0.759	0.881
4	CM	0.605	0.620	1.781	5.297
	DMLS	0.302	0.392	0.897	1.297
6.8	CM	0.162	0.166	0.179	0.183
	DMLS	0.031	0.035	0.038	0.044

**Table 3:** The released Cr from the CM and DMLS alloys (μg per cm^2^) in artificial saliva of pH = 2.3, pH = 4 and pH = 6.8.

pH	Sample	Time of the Exposure			
		1 Day	7 Days	14 Days	30 Days
2.3	CM	0.012	0.363	0.437	0.606
	DMLS	0.007	0.026	0.031	0.036
4	CM	0.011	0.033	0.061	0.209
	DMLS	0.003	0.006	0.005	0.002
6.8	CM	0.004	0.029	0.034	0.041
	DMLS	0.000	0.000	0.000	0.001

**Figure 8:**
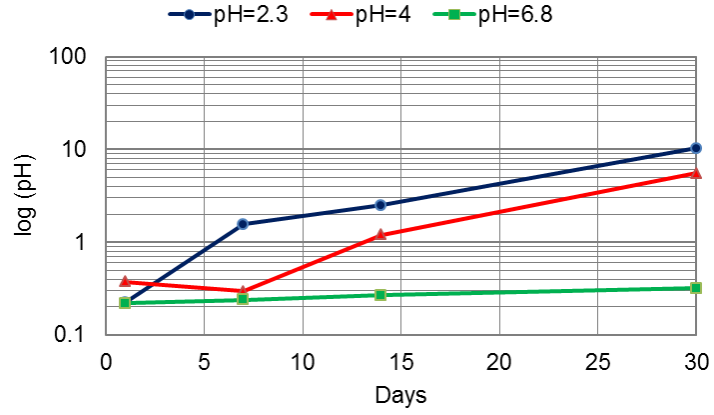
The difference between total release of Co, Cr and Mo from the CM and DMLS alloys (μg per cm^2^) in artificial saliva of pH = 2.3, pH = 4 and pH = 6.8.

The total release of metals in artificial saliva of different pH is presented in Table 5 and Figure 8. After seven days of immersion, the total release of Co, Cr and Mo from the CM alloy was three-times higher compared to the release from the DMLS alloy at a pH of 2.3, though both values were far below 200 μg per cm^2^, the limit prescribed by the ISO standard. In saliva of pH 2.3 and pH 4.5, the longer the investigated period, the higher the difference between the total metal ion release from the CM and DMLS alloys.

**Table 4:** The released Mo from the CM and DMLS alloys (μg per cm^2^) in artificial saliva of pH = 2.3, pH = 4 and pH = 6.8.

pH	Sample	Time of the Exposure			
		1 Day	7 Days	14 Days	30 Days
2.3	CM	0.059	0.279	1.133	2.431
	DMLS	0.015	0.061	0.093	0.113
4	CM	0.077	0.092	0.330	1.417
	DMLS	0.014	0.051	0.073	0.073
6.8	CM	0.092	0.113	0.130	0.179
	DMLS	0.092	0.008	0.034	0.039

**Table 5:** Total release of Co, Cr and Mo from the CM and DMLS alloys (μg per cm^2^) in artificial saliva of pH = 2.3, pH = 4 and pH = 6.8.

pH	Sample	Time of the exposure			
		1 Day	7 Days	14 Days	30 Days
2.3	CM	0.571	2.327	3.389	11.281
	DMLS	0.346	0.772	0.883	1.030
4	CM	0.693	0.745	2.172	6.923
	DMLS	0.319	0.449	0.975	1.372
6.8	CM	0.258	0.308	0.343	0.403
	DMLS	0.039	0.069	0.077	0.085

The release of all investigated metal ions was influenced by the acidity. The greatest release was measured in the most acidic environment. A change in acidity from pH of 6.8 to pH of 2.3 for the cast alloy led to an increase of three- to 45-times for Co, three- to 15-times for Cr and up to 13-times for Mo. The increase for the DMLS alloy was higher: eight- to 25-times for Co, up to 18-times for Cr and 1.8- to 2.8-times for Mo. The greatest release out of Co, Cr and Mo was for Co. Further, the greatest release of all ions was measured at pH 2.3 after 30 days of immersion (Table 2, Table 3 and Table 4). When comparing two technological procedures used to produce CM and DMLS samples, the greatest difference between the released ions occurred in the most acidic environment, where DMLS samples showed superior properties (Figure 5, Figure 6, Figure 7 and Figure 8). The presented results are in accordance with the results of Alifui-Segbaya *et al.* [17], *et al.* [12], as well as of Vandenbroucke and Kruth [31], who reported a lower release of metal ions from RM samples than from the cast Co-Cr-Mo alloy in the environment of pH 2.3. As there is no report on the influence of the acidity on the release of Co, Cr and Mo from the DMLS alloy, it is not possible to compare the results presented here with the results of other investigations.

The results of density analysis are presented in Table 6. The density of DMLS samples was found to be higher than for CM samples, about 4.5% on average. This is in line with research results of AM Co-Cr-Mo alloys reported by Vandenbroucke and Kruth [31] and Monroy *et al.* [32].

**Table 6:** Results of density analysis.

Sample	Density ρ (g/cm^3^)				
	ρ_average_	ρ_median_	ρ_stdev_	ρ_min._	ρ_max._
CM	8.22	8.22	0.02	8.20	8.24
DMLS	8.60	8.59	0.03	8.52	8.66

**Figure 9:**
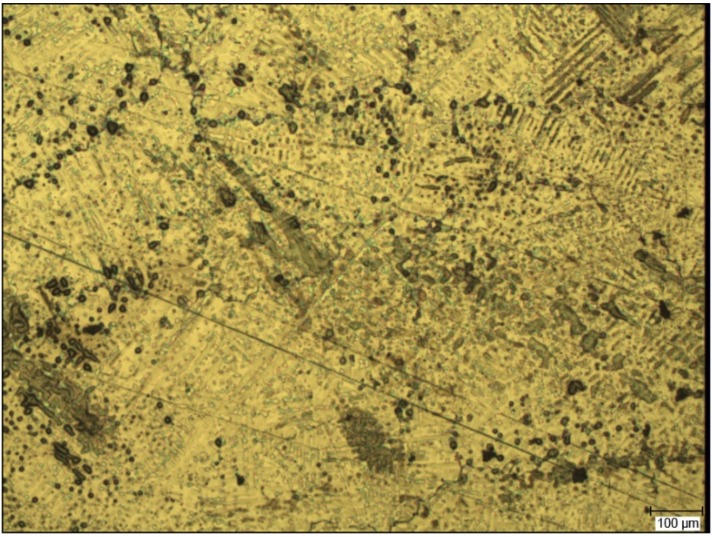
Microstructure of a cross-section of the CM sample. Magnification: 60×.

The microstructure of the CM alloy is shown in Figure 9, with the characteristic dendrite microstructure (dark portion in the figure), formed due to the different solidification temperatures of Co and Cr. The intermetallic phase varies in size and distribution, and it is presented by the light portion in the figure. Carbide precipitates can be seen as dark grey points of different sizes and shapes in the intermetallic phase, while endogenous porosity can be seen as black points.

**Figure 10:**
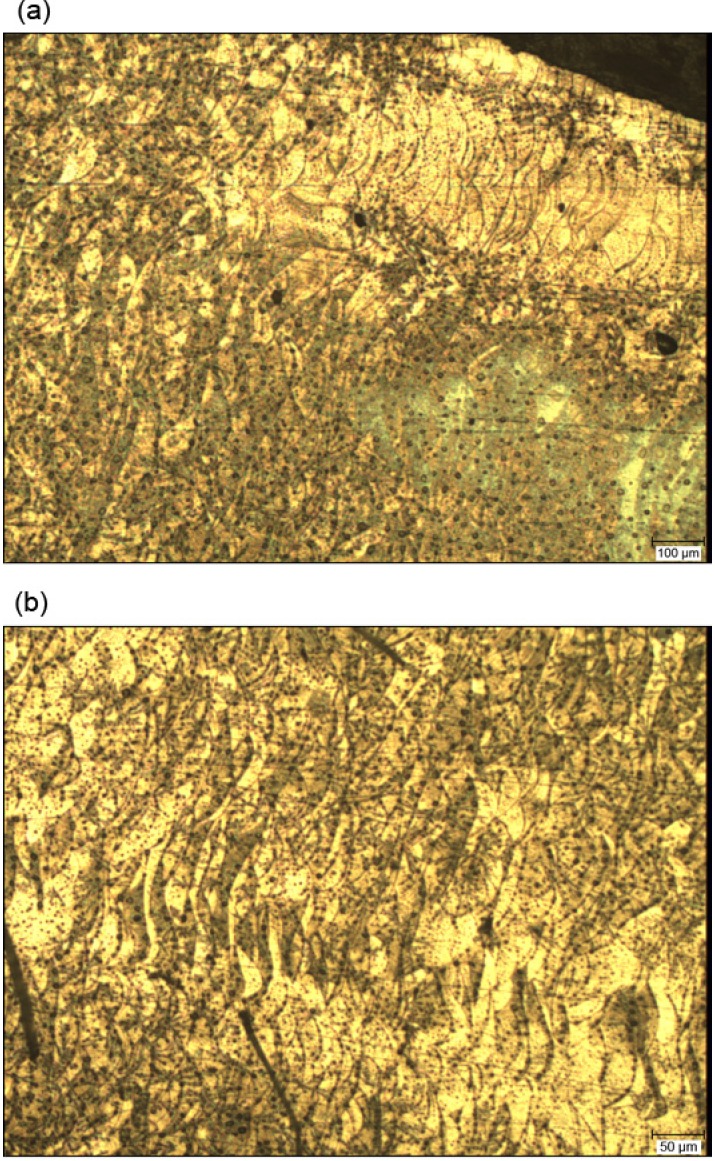
The microstructure of the cross-section of the DMLS sample: (**a**) magnification of 60×; (**b**) magnification of 120×.

The microstructure of the DMLS alloy (Figure 10) was observed in the transverse cross-section along the building direction of the DMLS process. The DMLS sample exhibited a more homogeneous structure than the CM sample. The fine microstructure indicates that metal particles melt evenly and sufficiently. In Figure 10a (magnification of 60×) and even better in Figure 10b (magnification of 120×), crystals can be seen as dark black points that are imbibed in yellow brownish intermetallic compounds. The bright yellow colour can be seen in a form of circular arch-shaped boundaries and waves, which correspond to laser scan tracks, or so-called melt pools, and they seem to be all oriented in the same direction. This is typical of the building direction of the DMLS process. The homogeneous microstructure of AM Co-Cr-Mo alloy is in accordance with the results of other corresponding investigations [12,32,33].

Considering the results of the metals’ dissolution analysis, higher density and a more homogeneous microstructure can be correlated to a lower metal elution, *i.e.*, to better corrosion resistance.

The elution of Co from dental devices made of Co-Cr-Mo alloy should be suppressed, as Co is known as a toxic and carcinogenic agent in higher concentrations [14]. Using DMLS technology, the release of Co can be significantly decreased. The results presented indicate the superior behaviour of the alloy made by this technology.

The results show that the release of Co, Cr and Mo from the DMLS alloy was lower than the release of ions from the CM alloy in artificial saliva of all investigated pH concentrations. The results show that the characteristics of the cast Co-Cr-Mo alloy are less favourable than the DLMS alloy in all tests. The corrosion characteristics of the cast Co-Cr-Mo alloy are well documented, as it has been used for decades for the manufacturing of dental devices [11]. Its apposite biological properties originate from the formation of a surface oxide layer consisting of oxides of Co and Cr [34].

There are a number of reasons why DMLS alloys demonstrate improved performance. The increased corrosion resistance of the DMLS alloy could be due to the addition of tungsten (W), which is known to improve the corrosion properties of Co-Cr alloys and to reduce chromium-depleted inter-metallic areas [35]. Furthermore, DMLS manufacturing is a completely different technology compared to conventional casting. While the casting procedure is difficult to control, the DMLS process parameters are computer controlled and, therefore, more repeatable. Finally, materials melted by laser show better homogeneity [31].

The average pH of human saliva is 6.8 [25], but in the oral cavity, pH can decrease even to 2.3. Acid environments in the oral cavity can be found in the small entrapped spaces near the dental devices, in the case of gastric juice regurgitation in the patients’ mouth, or it can be caused by repeated vomiting in pregnant women, or in the case of other gastrointestinal disorders, like bulimia. For investigating the influence of pH variations on the metal release, the most acidic (pH 2.3), the average (almost neutral pH 6.8) and one in between value (pH 4) were chosen.

According to the ISO standard mentioned above, the release of metallic ions over a seven-day immersion must be below 200 μg per cm^2^ of the tested sample under prescribed conditions [27]. The results of the investigation presented here show much lower amounts of ion release. The significantly lower release that occurred from the DMLS samples compared to cast samples can be seen in light of the present knowledge of the role of Co and Cr incorporated in cell metabolism, with oxidative stress playing a crucial role. Saliva is continuously excreted and swallowed within the oral cavity, so the released ions travel further through the digestive system. As these ions pass through the epithelial barrier in the intestines, it is possible that they can affect other organs and may have a systemic effect [15]. Although there is evidence that the passive oxide layer increases in passivity over time [17], a question arising is how much Co, Cr and Mo is released and swallowed by a patient wearing a partial denture metal alloy framework for five to 10 years. From that point of view, the results of many times lower release of metals from DMLS alloys may have more significance. This issue could be considered by future investigations. The measured quantity of the released ions indicate that both the technological procedures of casting and DMLS building of Co-Cr-Mo alloy devices are safe for use in the oral cavity according to the ISO standard, although encouragingly, DMLS specimens released many times less Co, Cr and Mo than CM specimens. The superiority of the DMLS alloy increases with the decrease of pH of the solution. The greater corrosion resistance of the DMLS-built alloy shows an excellent potential for dental applications.

## 4. Conclusions

In this study, the procedure and results of the investigation of ion release from Co-Cr-Mo dental alloys used by advanced DMLS technology and by conventional casting techniques were presented. The key difference between this and previous related studies is that the ion release was investigated in artificial saliva of different acidity, namely pH values of 6.8, 4 and 2.3.

Based on the results obtained, the following concluding remarks may be stated:

- Metal elution in artificial saliva from the DMLS alloy was lower than the elution from the cast alloy;
- Cobalt produced the greatest release of ions;
- Acidity influenced the elution;
- The greatest elution occurred in the most acidic environment, *i.e.*, 2.3 pH;
- The longer the investigated period, the higher the difference between the total metal ion release from the CM and DMLS alloys;
- Both alloys (CM and DMLS) showed a safe level of elution according to the ISO definition in all investigated acidic environments.

Future research, as mentioned above, could be focused on the long-term investigation of exposure to metal ions with patients wearing RPD made from Co-Cr-Mo alloys.
